# Expression levels of *HMGA2 *in adipocytic tumors correlate with morphologic and cytogenetic subgroups

**DOI:** 10.1186/1476-4598-8-36

**Published:** 2009-06-09

**Authors:** Hammurabi Bartuma, Ioannis Panagopoulos, Anna Collin, Domenico Trombetta, Henryk A Domanski, Nils Mandahl, Fredrik Mertens

**Affiliations:** 1 Department of Clinical Genetics, Lund University Hospital, Lund, Sweden; 2 Department of Genetics and Microbiology, University of Bari, Bari, Italy; 3 Department of Cytology and Pathology, Lund University Hospital, Lund, Sweden

## Abstract

**Background:**

The *HMGA2 *gene encodes a protein that alters chromatin structure. Deregulation, typically through chromosomal rearrangements, of *HMGA2 *has an important role in the development of several mesenchymal neoplasms. These rearrangements result in the expression of a truncated protein lacking the acidic C-terminus, a fusion protein consisting of the AT-hook domains encoded by exons 1–3 and parts from another gene, or a full-length protein; loss of binding sites for regulatory microRNA molecules from the 3' untranslated region (UTR) of *HMGA2 *has been suggested to be a common denominator.

**Methods:**

Seventy adipocytic tumors, representing different morphologic and cytogenetic subgroups, were analyzed by qRT-PCR to study the expression status of *HMGA2*; 18 of these tumors were further examined by PCR to search for mutations or deletions in the 3'UTR.

**Results:**

Type (full-length or truncated) and level of expression varied with morphology and karyotype, with the highest levels in atypical lipomatous tumors and lipomas with rearrangements of 12q13-15 and the lowest in lipomas with 6p- or 13q-rearrangements, hibernomas, spindle cell lipomas and myxoid liposarcomas. All 18 examined tumors showed reduced or absent expression of the entire, or parts of, the 3'UTR, which was not due to mutations at the DNA level.

**Conclusion:**

In adipocytic tumors with deregulated *HMGA2 *expression, the 3'UTR is consistently lost, either due to physical disruption of *HMGA2 *or a shift to production of shorter 3'UTR.

## Background

HMGA2 belongs to the high-mobility group A family of non-histone chromosomal proteins. With its three AT-hooks it can bind to the minor groove of AT-rich regions of DNA, affecting the transcription of target genes by modulating the DNA structure and facilitating or inhibiting the organization of enhanceosomes [1-3]. The HMGA2 protein has been shown to have important functions in cellular growth and differentiation, with a particular impact on the progression of the undifferentiated mesenchyme during fetal development [4]. *Hmga2 *knockout mice display reduced body weight with decreased fat levels as well as impaired fibroblast proliferation, whereas transgenic mice expressing activating mutations show somatic overgrowth with gigantism and lipomatosis [5-7]. Similarly, a boy with a constitutional chromosomal rearrangement resulting in deregulation of *HMGA2 *displayed a number of features indicative of disturbed mesenchymal differentiation, such as marked somatic overgrowth, skeletal abnormalities and multiple lipomas [8].

Limited studies on the expression patterns of the *HMGA2 *gene have shown that it is active in most tissues during fetal development, and that it is transcriptionally silent, or at least expressed at undetectable levels, in most adult differentiated tissues [9]. Aberrant deregulation, however, has been identified in a number of neoplasms, notably of mesenchymal origin [2]. In benign mesenchymal tumors, such as lipoma, the deregulation is often due to a cytogenetically visible rearrangement of the *HMGA2 *locus in 12q15, resulting in the expression of a truncated protein lacking the acidic C-terminus encoded by exons 4 and 5, a fusion protein consisting of the AT-hook domains encoded by exons 1–3 and more or less extensive parts from another gene, or, less commonly, expression of a full-length protein; apparently, all three forms – truncated, fusion and full-length – of the HMGA2 protein have transforming potential when expressed in the relevant cell type [6,7,10-15].

The loss of the C-terminal domains was long thought to be the cause of neoplastic transformation, but the finding that some tumor-associated translocations disrupt the 3'-untranslated region (3'-UTR) instead of the open reading frame (ORF), resulting in expression of the full-length HMGA2 protein, suggested that separation of repressive sequences in the 3'-UTR from the 5'-part of the gene could be an important mechanism behind transcriptional up-regulation [12,15,16]. Indeed, the 3'-UTR of *HMGA2 *is known to contain multiple binding sites for the *let-7 *family of microRNAs, small non-coding RNAs that inhibit gene expression at the posttranscriptional level, and it was recently shown that targeted mutation of these binding sites as well as functional inactivation of *let-7 *results in up-regulation of *HMGA2 *by reducing *HMGA2 *mRNA degradation in the cytoplasm [17,18].

Approximately 75% of conventional lipomas harbor chromosomal rearrangements of 12q13-15, strongly indicating the involvement of *HMGA2 *in these cases [19]. The remaining lipomas display other aberrations, including recurrent translocations affecting band 6p21, which harbors the *HMGA1 *gene, deletions of 13q, and supernumerary ring chromosomes. The importance of *HMGA2 *expression in these lipomas and other lipomatous tumors, remains poorly investigated. Over-expression of full-length or truncated *HMGA2 *has been demonstrated in some lipomas without 12q-rearrangement, as well as in atypical lipomatous tumors, but no systematic analysis of the status of *HMGA2 *in different cytogenetic subsets of lipomas or in other lipomatous tumors has been performed. In the present study, we used fluorescence in situ hybridization (FISH) and quantitative RT-PCR (qRT-PCR) to study the genomic status and expression level of full-length and truncated *HMGA2 *in various adipocytic tumors, including conventional lipomas, angiolipomas, spindle cell lipomas, hibernomas, atypical lipomas, well-differentiated liposarcomas, and myxoid liposarcomas. All cases expressing full-length *HMGA2*, as well as a few cases expressing truncated *HMGA2 *that served as controls, were further analyzed with regard to mutations in the 3'UTR.

## Methods

### Patients

A total of 73 adipocytic tumors were selected on the basis of their histopathologic diagnosis and/or cytogenetic profile. Due to lack of appropriate material, G-banding and expression analysis of *HMGA2 *could be performed in only 69 and 70 cases, respectively. Data concerning patient age and sex, location and karyotype are shown in Additional file 1. In brief, the study included five lipomas with translocation t(3;12)(q27;q13-15), five lipomas with t(5;12)(q32-33;q14-15), five lipomas with various other rearrangements of 12q14-15, five lipomas without any of the known specific cytogenetic hallmarks of lipoma, five lipomas with structural rearrangement of 6p21-22, five lipomas with deletion of chromosome arm 13q, five lipomas with ring chromosomes, eight angiolipomas, five spindle cell lipomas, five hibernomas, ten atypical lipomas, five well-differentiated liposarcomas, and five myxoid liposarcomas.

### Cytogenetic analyses

Culturing, harvesting, and chromosome banding of the tumor cells were performed as previously described [20]. Karyotypes were described according to ISCN (2009) [21]. The karyotypes of 29 samples have been reported before [19,22-25].

### Metaphase FISH analyses

**Figure 1:**
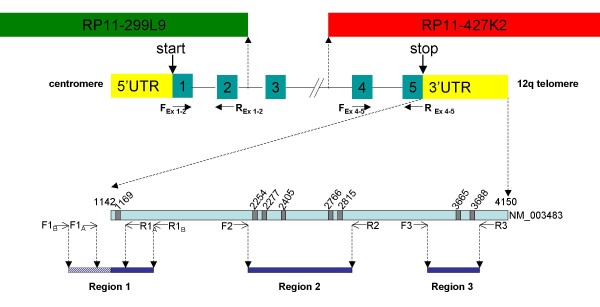
**Schematic illustration of the *HMGA2 *gene**. Locations of BAC clones RP11-299L9 (FITC) and RP11-427K2 (Cy3) and primers for the qRT-PCR reaction for exons 1–2 (**F_Ex 1–2_**: forward primer and **R_Ex 1–2_**: reverse primer) and exons 4–5 (**F_Ex 4-5_**: forward primer and **R_Ex 4-5_**: reverse primer) are indicated. The lower part shows an enlargement of the 3'UTR, where the base pair positions are based on the nucleotide sequence of NM_003483. Reported (Lee and Dutta, 2007) binding sites for *let-7 *are shown as dark boxes. Regions 1, 2 and 3 represent the sequences amplified by PCR; the locations of forward (F) and reverse (R) primers for each region are indicated. For details regarding primers, see Additional file 3.

Metaphase FISH was performed on all 18 cases from which unstained metaphase spreads were available, to study the status of the *HMGA2 *gene. BAC clones RP11-299L9 and RP11-427K2 that span the 5' and 3' ends, respectively, were used (Figure 1). Five cases had been analyzed previously [19,25]. Probes were selected through the UCSC Human Genome Browser genomic maps . Slides were prepared and analyzed and probes labeled as described elsewhere [26]. When applicable, whole chromosome painting probes (Applied Spectral Imaging, Migdal Haemek, Israel) were used to ensure that tumor cells were analyzed.

### DNA and RNA extraction

Total DNA from frozen tumor tissue and peripheral blood kept at -80°C was extracted using the DNeasy Blood and Tissue kit according to the manufacturer's recommendations (QIAGEN, Hilden, Germany). Total RNA from the same tumor tissue was extracted using the RNeasy Lipid Tissue Mini kit according to the manufacturer's recommendations (QIAGEN) with minor modifications; 400 mg of tissue were used instead of the recommended 100 mg. After transferring the upper aqueous phase to a new collection tube, it was spun down once more at 12 000 × g for 15 min at 4°C.

### cDNA synthesis

Three micrograms of total RNA were reverse transcribed in a volume of 50 μl for cDNA synthesis, as described previously [19]. Fetal cDNA from kidney, liver, lung, brain, heart, spleen, thymus and skeletal muscle (Clontech, Moutain View, California, USA) were used for RT-PCR.

### Quantitative RT-PCR analyses (qRT-PCR)

qRT-PCR was carried out to determine the expression level of *HMGA2*. The *HMGA2 *TaqMan gene expression assays (Applied Biosystems, Foster City, CA, USA) Hs00171569_m1 (*HMGA2 *exons 1–2) and Hs00971725_m1 (*HMGA2 *exons 4–5) were used for the qRT-PCR reaction. Human *ACTB *was used as endogenous control for normalization [Human ACTB (beta actin) Endogenous Control FAM/MGB Probe, Non-Primer Limited, Applied Biosystems]. To ensure reliable results three replicates of each sample and endogenous control were performed. For the commercial assay we had 1×TaqMan universal Mix, l× of the 20×TaqMan gene expression mix and 3 μl cDNA, in a volume of 20 μl. The PCR was run on a 7500 real-time PCR System (Applied Biosystems) using the standard 7500 run mode. The PCR conditions were 2 min at 50°C and 10 min at 95°C, followed by 50 cycles at 95°C for 15 sec, and 1 min at 60°C. The SDS software 1.3.1 (Applied Biosystems) was used to analyze data. The comparative threshold cycle (Ct) method was used to achieve relative quantification of RNA expression [27]: the value of the target, normalized to an endogenous control (ACTB) and relative to a calibrator, was expressed as 2^-ΔΔCt ^(fold difference), where ΔCt of the target gene samples minus the ΔCt of the target gene calibrator gives the ΔCt value of the target message [27]. Total RNA from human adipose tissue (Ambion's total Human RNA, Austin, TX, USA) was the calibrator for cDNA control. Expression cycles for exons 1–2 and 4–5 of *HMGA2 *in human adipose tissue are illustrated, see Additional file 2.

### Genomic PCR, RT-PCR and Sequence analyses

PCR and sequencing were performed to study cryptic deletions and point mutations in the *HMGA2 *3'UTR, harboring the target sites of the *let-7 *miRNA family (Figure 1), in 18 tumors. These cases included all nine tumors with full-length *HMGA2 *expression and from which further material was available, as well as controls showing differential or weak expression of *HMGA2*. The PCR reaction was performed as described [19]. The PCR products were analyzed on 2–2.5% agarose gels. Primers used for sequencing are listed in Additional file 3. All transcripts identified were verified by sequencing, and the corresponding nucleotide sequences were analyzed using the Chromas software . The *HMGA2 *origin of the sequences was verified by BLAST search . Sequence data on *HMGA2 *were obtained from the sequence of the clone with accession number NM_003483 [GenBank:NM_003483]. Polymorphisms were identified from the ensembl database .

## Results

### Cytogenetics and FISH

**Figure 2:**
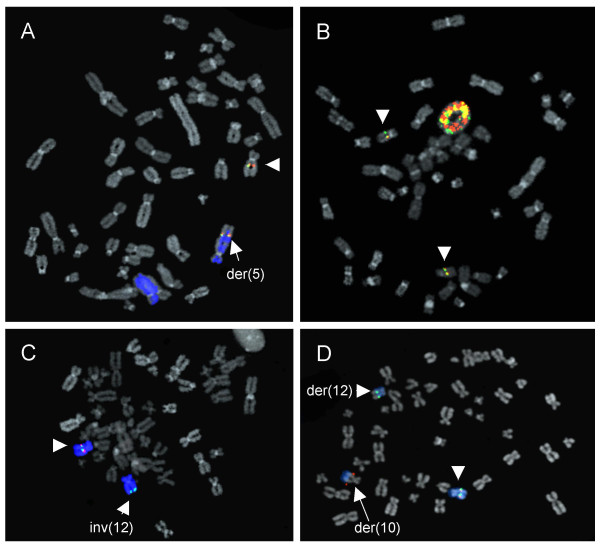
**Metaphase FISH on four cases regarding the involvement of the *HMGA2 *gene**. BAC clones RP11-299L9 (FITC) and RP11-427K2 (Cy3), spanning the 5' and 3' parts, respectively, of *HMGA2 *were used. Arrowheads indicate normal chromosomes 12 **A) **Lipoma (case 10) with a t(5;12)(q33;q14) where the entire *HMGA2 *locus was translocated to chromosome 5. A wcp5 probe was used to identify chromosome 5 in tumor cells. The derivative chromosome 12 was present in the metaphase cell, but not included in the picture **B) **Atypical lipoma (case 55) with ring chromosome and amplification and split signal of *HMGA2*. Normal signals for *HMGA2 *can be seen on the two normal chromosomes 12. **C) **Lipoma (case 11) with an inv(12)(q14q24) causing a deletion of the 3'-end of *HMGA2*; thus, only the green signal corresponding to RP11-299L9, covering the 5'-part, can be seen. **D) **Lipoma (case 14) with a t(10;12)(q22;q15) resulting in a split signal for *HMGA2*; the 3' part of the gene is translocated to chromosome 10. A wcp12 was used to identify chromosome 12 in tumor cells.

Sixty-nine of the 73 cases were analyzed by G-banding; from four of the cases no short-term cultures were available. Metaphase FISH analysis regarding the status of the *HMGA2 *gene was performed on 18 cases (Additional file 1). In brief, of four analyzed lipomas with t(5;12)(q32-33;q14-15), two displayed normal signals for the *HMGA2 *gene, one showed a split signal, and one showed translocation of the entire *HMGA2 *gene to chromosome 5 (Figure 2A). Of four lipomas with various aberrations affecting 12q13-15, three had split signals and one had a deletion of the 3'-end of the gene (Figure 2C and 2D). Of three lipomas without any of the recognized cytogenetic hallmarks of lipoma two had normal signals and one a deletion of the 3'-end of the gene. The only analyzed lipoma with a translocation involving 6p21-22 and two angiolipomas had normal signals for *HMGA2*. Four atypical lipomas with ring chromosomes showed amplification of *HMGA2*, with more copies of the 5'-end than of the 3'-end; intact signals were consistently found on the normal chromosomes 12 (Figure 2B).

### qRT-PCR

**Figure 3:**
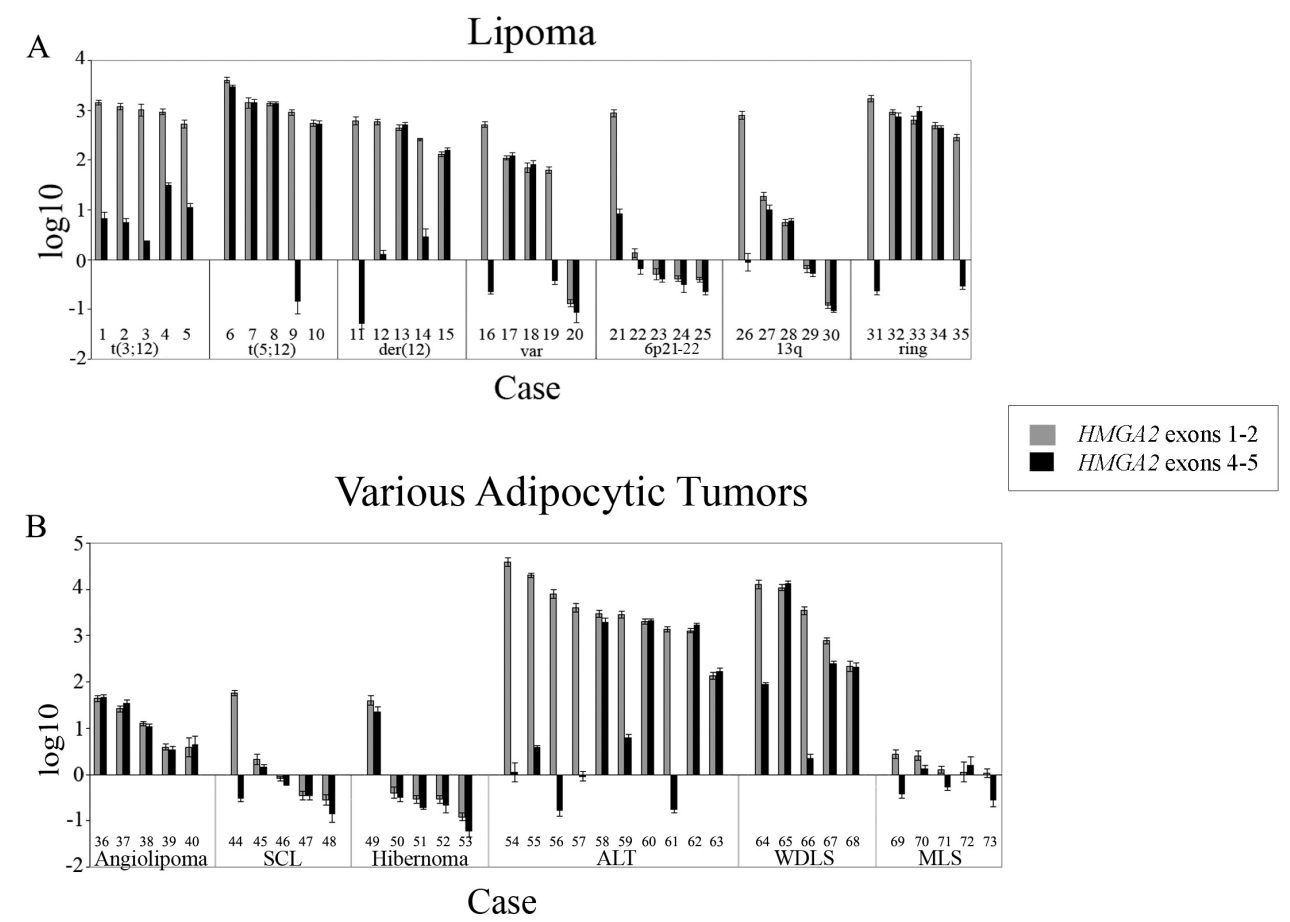
**Expression patterns (log10 scale) of exons 1–2 and exons 4–5 of *HMGA2 *in lipomatous tumors**. **A) **Seven different cytogenetic subgroups of conventional lipoma: lipomas with t(3;12)(q27;q13-15), lipomas with t(5;12)(q32-33;q14-15), lipomas with various other rearrangements of 12q14-15, lipomas without any of the known cytogenetic hallmarks of lipoma, lipomas with structural rearrangement of 6p21-22, lipomas with deletion of chromosome arm 13q, and lipomas with ring chromosomes. **B) **Six other subtypes of lipomatous tumor: angiolipomas, spindle cell lipomas, hibernomas, atypical lipomas, well-differentiated liposarcomas, and myxoid liposarcomas. The case numbers correspond to the case numbers in Additional file 1.

A total of 70 adipocytic tumors were investigated with qRT-PCR for the expression of *HMGA2 *exons 1–2 and exons 4–5. Aberrant expression of *HMGA2 *was defined as a Log10 value of > 1 for exons 1–2 and/or 4–5; expression levels > 10 times higher for exons 1–2 than for exons 4–5 are hereafter referred to as differential expression. Using these criteria, aberrant expression was seen in 47 cases, of which 24 showed differential expression (detailed results in Additional file 1). None of the cases showed aberrant expression of exons 4–5 without simultaneous aberrant expression of exons 1–2. In brief, the expression levels of exons 1–2 were highest among well-differentiated liposarcomas (median Log10 value of 3.55), followed by atypical lipomas (3.46), t(5;12)-lipomas (3.13), t(3;12)-lipomas (3.00), conventional lipomas with ring chromosomes (2.80), lipomas with various rearrangements of 12q13-15 (2.65), lipomas without any of the recognized cytogenetic hallmarks of lipoma (1.85), angiolipomas (1.10), del(13q)-lipomas (0.74), lipomas with rearrangements of 6p21 (0.13), myxoid liposarcomas (0.11), spindle cell lipomas (-0.09), and hibernomas (-0.53). Individual expression levels are illustrated in Figure 3.

With regard to the number of cases showing aberrant and differential expression, the results were as follows: of 15 lipomas with cytogenetic rearrangement of 12q13-15 all showed aberrant expression, with differential expression in all five lipomas with t(3;12), one of five lipomas with t(5;12)(q32-33;q14-15), and three of five lipomas with various other rearrangements of 12q13-15. Four of five lipomas without any of the recognized cytogenetic hallmarks of lipoma showed aberrant expression, with differential expression in two. One of five lipomas with rearrangement of 6p21-22 showed differential expression, and of five lipomas with del(13q), two showed aberrant expression with differential expression in one. All 20 lipomatous tumors with ring chromosomes showed aberrant expression, with differential expression in two of five conventional lipomas, six of ten atypical lipomas, and two of five well-differentiated liposarcomas. Three of five angiolipomas showed aberrant expression, without any sign of differential expression. None of the five myxoid liposarcomas and only one case each of spindle cell lipoma (differential expression) and hibernoma (full-length) showed aberrant expression.

### RT-PCR and genomic PCR

The expression of the reported 3'UTR of *HMGA2*, divided into three regions covering all eight known binding sites for *let-7*, was studied by RT-PCR in 18 tumors, various fetal tissues and amniocytic cell cultures (Table 1). Of the 18 tumors, nine had aberrant expression of both exons 1–2 and 4–5, four showed differential expression of exons 1–2, two showed weak expression of both exons 1–2 and 4–5, and three had unknown expression status. All three amniocytic cell cultures expressed all three regions of the 3'UTR (see Additional file 4A). In the fetal tissues, expression of Region 1 was found in kidney, liver, lung, spleen, and thymus, but the bands were weak except in kidney. Expression of regions 2 and 3 was seen in all tissues except brain, which also lacked expression of region 1 (see Additional file 5A). Of the tumors, only one (case 10) expressed all three regions, but with very weak expression of region 2. Faint bands for region 1 and/or 3 were seen in nine tumors. In the remaining eight tumors, including two cases with differential expression of *HMGA2*, none of the regions was detected (Table 1, see Additional file 4A). Genomic PCR for the 3'UTR of *HMGA2 *was performed in 15 of the tumors that had been studied by RT-PCR; in addition, DNA from peripheral blood was available from six of the cases. PCR products of expected size were seen in all tumors and blood samples (see Additional files 4B and 5B).

**Table 1:** RT-PCR and genomic PCR analyses of the 3' UTR of *HMGA2*

			**RT-PCR products Region**			**Genomic PCR products Region**		
**Case**	**Sample**	**Type of *HMGA2 *expression**	**1**	**2**	**3**	**1**	**2**	**3**
	Amniocytes 1	ND	+	+	+	ND	ND	ND
	Amniocytes 2	ND	+	+	+	ND	ND	ND
	Amniocytes 3	ND	+	+	+	ND	ND	ND
4	Lipoma t(3;12)	Differential	(+)	-	(+)	+	+	+
4	Peripheral blood	ND	ND	ND	ND	+	+	+
10	Lipoma t(5;12)	Aberrant	+	(+)	+	+	+	+
11	Lipoma der(12q13-15)	Differential	-	-	-	+	+	+
13	Lipoma der(12q13-15)	Aberrant	-	-	-	+	+	+
17	Variant Lipoma	Aberrant	(+)	-	(+)	+	+	+
18	Variant Lipoma	Aberrant	(+)	-	-	+	+	+
18	Peripheral blood	ND	ND	ND	ND	+	+	+
21	Lipoma der(6p21-22)	Differential	-	-	(+)	ND	ND	ND
26	Lipoma del(13q)	Differential	-	-	-	+	+	+
26	Peripheral blood	ND	ND	ND	ND	+	+	+
27	Lipoma del(13q)	Aberrant	-	-	-	ND	ND	ND
36	Angiolipoma	Aberrant	(+)	-	+	ND	ND	ND
37	Angiolipoma	Aberrant	-	-	(+)	+	+	+
38	Angiolipoma	Aberrant	-	-	(+)	+	+	+
38	Peripheral blood	ND	ND	ND	ND	+	+	+
39	Angiolipoma	Weak	-	-	-	+	+	+
39	Peripheral blood	ND	ND	ND	ND	+	+	+
40	Angiolipoma	Weak	-	-	-	+	+	+
40	Peripheral blood	ND	ND	ND	ND	+	+	+
41	Angiolipoma	ND	-	-	-	+	+	+
42	Angiolipoma	ND	-	-	(+)	+	+	+
43	Angiolipoma	ND	-	-	-	+	+	+
49	Hibernoma	Aberrant	-	-	(+)	+	+	+

Abbreviations: Aberrant = Log10 value of ≥ 1 for exons 1–2 and exons 4–5; Differential = Aberrant expression of exons 1–2 with ≥ 10 times higher expression than for exons 4–5; Weak = Log10 value 0–1 for exons 1–2 and 4–5; + = strong PCR product; (+) = weak PCR product; - = no PCR product; ND = not determined.

### Sequencing

Sequencing of the 3'UTR of *HMGA2 *was performed on the 15 tumors in which genomic PCR was conducted to study involvement of the *let-7 *binding sites. As reference, the sequence with accession number NM_003483 was used [GenBank NM_003483]. In region 1, eight of the 15 tumors were homozygous for G/G, six homozygous for T/T and one heterozygous for T/G for a previously reported polymorphism in position 1280 (reference number rs3741427). In region 2, one tumor was homozygous for C/C, two were homozygous for T/T, and 12 were heterozygous for C/T at position 2416 (reference number rs1042725). In region 3, no mutations were seen.

## Discussion

Among the 35 conventional lipomas that were analyzed here, four expression patterns were observed: (1) strong (>100-fold) expression of the full-length gene (exons 1–2 as well as 4–5) or (2) a truncated gene (only exons 1–2; differential expression), (3) moderate (10–100-fold) expression of the entire gene, and (4) weak/absent expression of any part of the gene. Although we cannot fully exclude that low expression in some case was due to admixture of normal cells, it is of interest to note that the type of expression pattern varied with cytogenetic subgroup. All five lipomas with t(3;12) showed strong (log10 ratio between 2.72 and 3.15), differential expression. The t(3;12) is the most common cytogenetic aberration in conventional lipoma, resulting in an *HMGA2/LPP *fusion gene. In cases reported in the literature, the fusion gene always contains exons 1–3 of *HMGA2 *and, usually, exons 9–11 of *LPP*, which is at odds with the finding of increased (log10 ratios 1.04 and 1.49) expression of exons 4–5 in two of our cases. However, a few reported lipomas with t(3;12) have also expressed the reciprocal *LPP/HMGA2 *chimera and some tumors with rearrangements of *HMGA2 *express the wild type allele [14,28-31].

In contrast, only one of five lipomas with a t(5;12), the second most common translocation in conventional lipomas, showed differential expression in the present study; the other four cases all showed aberrant expression of the entire gene. This is in line with previous FISH data on this particular translocation, demonstrating that the genomic breakpoints usually occur outside the *HMGA2 *locus [25]. The only case here with differential expression also had a split *HMGA2 *locus at FISH analysis, supporting the view that the molecular outcome of the t(5;12) is heterogeneous. Cytogenetic analyses of conventional lipomas have identified numerous other translocation partners to the *HMGA2 *locus in 12q [19]. Although only a few of these partners have been examined at the molecular level, it is well known from FISH studies that the breakpoints may occur inside as well as outside *HMGA2*. Thus, it was not unexpected that all five lipomas with variant 12q13-15 rearrangements in the present study, representing five different recombination partners, showed overexpression of *HMGA2 *and that some of them had expression of the full-length gene whereas others had differential expression. Interestingly, strong expression of the full-length gene was seen in one case with a t(1;12)(p32;q15) and a split FISH signal, with the 3'-part of *HMGA2 *translocated to chromosome 1. Combined, these findings indicate that this particular translocation led to the fusion of *HMGA2 *with a strongly expressed gene in chromosome 1. However, high expression of the other, wild type, allele cannot be excluded.

The molecular pathways in conventional lipomas with rearrangements of 6p or deletions of 13q remain to be clarified. It is commonly believed that rearrangements of the *HMGA1 *gene, located in band 6p21, could substitute for *HMGA2 *rearrangements, but this has been verified in only a small number of cases, [19,32-34] and no candidate target for 13q-deletions has been identified. The notion that the molecular pathogenesis of these lipomas is different from that in 12q-positive ones was strengthened by the finding here that only one case in each group showed strong *HMGA2 *expression. Further, indirect support for the hypothesis that most lipomas with 6p21 or 13q-rearrangements develop through pathways that do not involve *HMGA2 *could be found among the lipomas that showed aberrations that do not belong to any of the known cytogenetic subgroups of conventional lipomas; four of five showed aberrant (log10 ratio 1.80–2.17) expression of the 5'part or of the entire gene. Thus, most of the non-recurrent cytogenetic aberrations observed are probably secondary changes, accrued during tumor growth.

Ring chromosomes and giant markers (hereafter referred to as rings) constitute the cytogenetic hallmark of atypical lipomatous tumors, and it has been shown that these rings consistently contain amplified sequences from chromosome 12 [35-38]. Occasionally, also tumors diagnosed as conventional lipoma display rings, and it has been debated whether these cases represent highly differentiated atypical lipomatous tumors or whether some are true conventional lipomas with a similar genetic profile [19,36,39-42]. The former interpretation seems the more likely; not only do tumors diagnosed as conventional lipomas with rings share the clinical characteristics of atypical lipomatous tumors (male predominance, larger size, preferential location in the thigh), but the genomic contents of the rings are the same [42-44]. In the present study, the expression of *HMGA2 *was increased (full-length in three and differential in two) in all five cases diagnosed as conventional lipomas with rings. However, the expression levels (median log10 ratio for exons 1–2, 2.80) were more similar to those seen in lipomas with t(3;12) and t(5;12) (median log10 ratios 3.00 and 3.13, respectively) than in tumors diagnosed as atypical lipomas and well-differentiated liposarcomas (median log10 ratios 3.46 and 3.55, respectively), whereas the fraction of lesions with differential expression was similar in all three tumor entities. This raises the interesting possibility that the level of cellular atypia may be influenced by the level of *HMGA2 *expression in lipomatous tumors with rings. However, the rings are highly complex chromosome aberrations and it cannot be excluded that the amplification and expression levels of other genes influence the phenotype.

Whereas atypical lipomatous tumors in the extremities seldom recur after surgery and only rarely transform into high-grade lesions, morphologically and cytogenetically identical lesions in the retroperitoneum commonly dedifferentiate, eventually often killing the patient. To emphasize these differences in clinical behaviour, the former are often referred to as atypical lipomas, and the latter as well-differentiated liposarcomas. In the present study, we analyzed ten atypical lipomatous tumors of the extremities and five of the retroperitoneum. They consistently showed high expression of *HMGA2*, arguing for the importance of deregulated *HMGA2 *expression also in this subtype of lipomatous tumors [36,45].

Taken together, these results demonstrate that all 35 lipomatous tumors with cytogenetically visible 12q13-15 rearrangements – translocations, inversions, rings – have aberrant expression of *HMGA2*, and that some of these rearrangements, like the t(3;12), consistently result in differential expression of the first three exons whereas other rearrangements, like the t(5;12), most often lead to expression of the entire gene. The lack of *HMGA2 *expression in most cases with rearrangements of 6p21 or deletions of 13q strongly support the view that these lipomas develop through other genetic pathways. The consistently high expression of *HMGA2 *in conventional lipomas with ring chromosomes provides further support for the idea that these tumors belong to the same biologic entity as atypical lipomas and well-differentiated liposarcomas.

As expected, none of the five myxoid liposarcomas, which all had a t(12;16) resulting in a *FUS-DDIT3 *fusion gene (data not shown), expressed *HMGA2*. Similarly, most hibernomas and spindle cell lipomas, which at the chromosome level typically show translocations affecting chromosome band 11q13 and deletions of 13q, respectively, did not show any deregulation of *HMGA2*. However, one case each demonstrated moderate expression of the full-length gene (hibernoma, log10 ratio exons 1–2, 1.60) or only of the amino-terminal part (spindle cell lipoma, log10 ratio exons 1–2, 1.76). Although both cases were morphologically typical, it is well known that hibernomas and spindle cell lipomas may contain more or less extensive areas resembling conventional lipomas, presumably reflecting differentiation. Possibly, such areas might be associated with deregulated expression of *HMGA2*.

Angiolipomas often occur as multiple tumors, and an autosomal dominantly inherited predisposition is apparent in some families [46,47]. Repeated cytogenetic analyses have, with a single exception, resulted in normal karyotypes [23], suggesting that they are either non-neoplastic or that they arise through submicroscopic mutations [46]. In the present study, five cases could be analyzed with regard to expression level of *HMGA2*, two of which were also studied by FISH; none of the latter showed any cryptic rearrangement of the *HMGA2 *locus. In all cases, the expression levels were highly similar for exons 1–2 and 4–5, and the median expression level (log10 ratio 1.10 for exons 1–2) was higher than that in myxoid liposarcomas, hibernomas, spindle cell lipomas, and lipomas with 13q-deletions or 6p21-rearrangements, but lower than that in conventional lipomas with cytogenetically visible 12q-rearrangements. Thus, it seems as if also angiolipomas, at least to some extent, are associated with aberrant *HMGA2 *expression, but not on the basis of gross chromosomal rearrangements.

To study the role of the 3'UTR for deregulated *HMGA2 *expression, RT-PCR was performed in 18 lipomatous tumors, including nine with aberrant expression of the full-length gene, four with differential expression, and five with weak or unknown expression (Table 1). The 3'UTR of *HMGA2 *is 2996 bp long, according to the sequence reported in 1996 by Ashar et al. [GenBank:NM_003483][48]. It contains a number of potential termination sites before the one that ends the reported full-length UTR. In addition, there are 10 AUUUA elements, which are thought to be important for deadenylation and subsequent decay of the mRNA [15]. We are not aware of any previous, systematic *in vivo *studies of the expression of the 3'UTR, neither in tumors nor in non-neoplastic tissues, but several experimental studies have suggested that the 3'UTR plays an important role in the posttranscriptional regulation of *HMGA2*. In 2001, Borrmann et al. measured the luciferase activity of the full-length and five different deletion constructs of the *HMGA2 *3'UTR [15]. They found that truncation of the distal 593 bp reduced the activity, suggesting the existence of positive regulatory elements at the 3' end of the UTR, but that further deletions significantly increased the luciferase activity. This increase was interpreted to be due to the loss of the AUUUA elements. More recently, it was shown that the 3'UTR of *HMGA2 *contains eight, functional or putative, binding sites for the *let-7 *family of microRNAs, and that mutational inactivation of these binding sites increased the mRNA levels of *HMGA2 *[17,18,49,50]. In line with these findings, it is known that *let-7 *and *HMGA2 *expression levels are inversely correlated during embryogenesis. This agrees well with the strong expression of the entire 3'UTR in all three amniocytic cell cultures included here. In contrast, only two of the tumors, a lipoma with t(5;12) and a relocated *HMGA2 *locus and an angiolipoma, showed convincing expression of any of the three regions. The remaining tumors, regardless of morphologic subtype and type of *HMGA2 *expression, showed only faint expression of no more than two of the three regions investigated, providing further *in vivo *support for the notion that loss of regulatory sequences from the 3'UTR is essential for aberrant expression of *HMGA2 *in lipomatous tumors. However, as loss or significant reduction of expression of the 3'UTR was a consistent feature also of tumors with expression of the full-length gene and normal *HMGA2 *loci at FISH analysis, there must be other mechanisms than chromosomal rearrangements leading to this transcriptional silencing. It was recently shown by Sandberg et al. (2008) that proliferating cells tend to shift from producing longer to shorter mRNA isoforms, and they suggested that a switch to more proximal polyadenylation sites might be a mechanism for these cells to escape regulation by microRNAs [51]. How this shift from expressing mRNAs with full-length 3'UTR, as seen in the amniocytic cultures, to mRNAs without the 3'UTR, or at least with a 3'UTR too short to be detected by the assays used here, is achieved in lipomatous tumors remains to be clarified. Our and others results indicate that the expression of *HMGA2 *[9,52] as well as its 3'UTR varies from one tissue to another. Based on the present study, however, the lack of 3'UTR expression seems not to be due to mutations within the 3'UTR itself. Furthermore, nor could we find any evidence that a SNP (rs1042725) within the 3'UTR that has been associated with body height [53] has any impact on the development of lipomatous tumors.

## Conclusion

We have shown that the expression (level and/or type of transcript) of *HMGA2 *in adipocytic tumors varies with morphologic subtype and cytogenetic background. Thus, transcriptional deregulation was strongly associated with cytogenetically visible involvement of the *HMGA2 *locus. We also showed that tumors expressing *HMGA2 *do not express the 3'UTR of the gene, thereby avoiding down-regulation by the *let-7 *family of microRNAs.

## Competing interests

The authors declare that they have no competing interests.

## Authors' contributions

HB performed the FISH and PCR analyses, collected and analyzed the data and wrote the manuscript. IP and AC assisted in designing the experiments and interpreting the results. DT carried out part of the sequencing and analyses. HAD was responsible for the morphologic classification of the tumors. NM and FM were responsible for the cytogenetic data and for the design of the study. All authors have read and approved the final manuscript.

## Supplementary Material

Additional file 1 **Supplementary table**. Clinical, cytogenetic, qRT-PCR, and FISH Data on 73 adipocytic tumors Click here for file

Additional file 2 **Expression pattern of *HMGA2 *exons 1–2 and 4–5 in human adipose tissue**. **A) **exons 1–2 and **B) **exons 4–5 of *HMGA2 *in human adipose tissue [Ambion's total Human RNA] showing a late expression pattern, starting after 35 cycles in both amplifications. Three replicates were run. Click here for file

Additional file 3 **Supplementary table**. Primers for PCR Click here for file

Additional file 4 **RT-PCR and genomic PCR for the 3'UTR of *HMGA2***. RT-PCR and genomic PCR for the 3'UTR of *HMGA2 *in 3 amniocytic cell cultures and 18 adipocytic tumors. Click here for file

Additional file 5 **RT-PCR and genomic PCR for the 3'UTR of *HMGA2***. RT-PCR analysis of the 3'UTR of *HMGA2 *in fetal tissues and genomic PCR analysis of the 3'UTR of *HMGA2 *in DNA from peripheral blood from six of the patients with lipomatous tumors. Click here for file

## References

[B1] Reeves R, Beckerbauer L (2001). HMGI/Y proteins: flexible regulators of transcription and chromatin structure. Biochim Biophys Acta.

[B2] Fusco A, Fedele M (2007). Roles of HMGA proteins in cancer. Nat Rev Cancer.

[B3] Cleynen I, Ven WJ Van de (2008). The HMGA proteins: a myriad of functions. Int J Oncol.

[B4] Li O, Li J, Dröge P (2007). DNA architectural factor and proto-oncogene *HMGA2 *regulates key developmental genes in pluripotent human embryonic stem cells. FEBS Lett.

[B5] Zhou X, Benson KF, Ashar HR, Chada K (1995). Mutation responsible for the mouse pygmy phenotype in the developmentally regulated factor *HMGI-C*. Nature.

[B6] Battista S, Fidanza V, Fedele M, Klein-Szanto AJ, Outwater E, Brunner H, Santoro M, Croce CM, Fusco A (1999). The expression of a truncated *HMGI-C *gene induces gigantism associated with lipomatosis. Cancer Res.

[B7] Zaidi MR, Okada Y, Chada KK (2006). Misexpression of full-length *HMGA2 *induces benign mesenchymal tumors in mice. Cancer Res.

[B8] Ligon AH, Moore SD, Parisi MA, Mealiffe ME, Harris DJ, Ferguson HL, Quade BJ, Morton CC (2005). Constitutional rearrangement of the architectural factor *HMGA2*: a novel human phenotype including overgrowth and lipomas. Am J Hum Genet.

[B9] Gattas GJ, Quade BJ, Nowak RA, Morton CC (1999). *HMGIC *expression in human adult and fetal tissues and in uterine leiomyomata. Genes Chromosomes Cancer.

[B10] Ashar HR, Fejzo MS, Tkachenko A, Zhou X, Fletcher JA, Weremowicz S, Morton CC, Chada K (1995). Disruption of the architectural factor *HMGI-C*: DNA-binding AT hook motifs fused in lipomas to distinct transcriptional regulatory domains. Cell.

[B11] Schoenmakers EF, Wanschura S, Mols R, Bullerdiek J, Van den Berghe H, Van de Ven WJ (1995). Recurrent rearrangements in the high mobility group protein gene, *HMGI-C*, in benign mesenchymal tumours. Nat Genet.

[B12] Geurts JM, Schoenmakers EF, Van de Ven WJ (1997). Molecular characterization of a complex chromosomal rearrangement in a pleomorphic salivary gland adenoma involving the 3'-UTR of *HMGIC*. Cancer Genet Cytogenet.

[B13] Fedele M, Berlingieri MT, Scala S, Chiariotti L, Viglietto G, Rippel V, Bullerdiek J, Santoro M, Fusco A (1998). Truncated and chimeric *HMGI-C *genes induce neoplastic transformation of *NIH3T3 *murine fibroblasts. Oncogene.

[B14] Klotzbücher M, Wasserfall A, Fuhrmann U (1999). Misexpression of wild-type and truncated isoforms of the high-mobility group I proteins *HMGI-C *and *HMGI(Y) *in uterine leiomyomas. Am J Pathol.

[B15] Borrmann L, Wilkening S, Bullerdiek J (2001). The expression of *HMGA *genes is regulated by their 3'UTR. Oncogene.

[B16] Quade BJ, Weremowicz S, Neskey DM, Vanni R, Ladd C, Dal Cin P, Morton CC (2003). Fusion transcripts involving HMGA2 are not a common molecular mechanism in uterine leiomyomata with rearrangements in 12q15. Caner Res.

[B17] Lee YS, Dutta A (2007). The tumor suppressor microRNA *let-7 *represses the *HMGA2 *oncogene. Genes Dev.

[B18] Mayr C, Hemann MT, Bartel DP (2007). Disrupting the pairing between *let-7 *and *Hmga2 *enhances oncogenic transformation. Science.

[B19] Bartuma H, Hallor KH, Panagopoulos I, Collin A, Rydholm A, Gustafson P, Bauer HCF, Brosjö O, Domanski HA, Mandahl N, Mertens F (2007). Assessment of the clinical and molecular impact of different cytogenetic subgroups in a series of 272 lipomas with abnormal karyotype. Genes Chromosomes Cancer.

[B20] Mandahl N, Heim S, Arheden K, Rydholm A, Willén H, Mitelman F (1988). Three major cytogenetic subgroups can be identified among chromosomally abnormal solitary lipomas. Hum Genet.

[B21] Shaffer LG, Slovak ML, Campbell LJ, ISCN (2009). An International System for Human Cytogenetic Nomenclature 2009.

[B22] Heim S, Mandahl N, Kristoffersson U, Mitelman F, Rööser B, Rydholm A, Willén H (1987). Marker ring chromosome – a new cytogenetic abnormality characterizing lipogenic tumors?. Cancer Genet Cytogenet.

[B23] Mandahl N, Höglund M, Mertens F, Rydholm A, Willén H, Brosjö O, Mitelman F (1994). Cytogenetic aberrations in 188 benign and borderline adipose tissue tumors. Genes Chromosomes Cancer.

[B24] Dahlén A, Debiec-Rychter M, Pedeutour F, Domanski HA, Höglund M, Bauer HC, Rydholm A, Sciot R, Mandahl N, Mertens F (2003). Clustering of deletions on chromosome 13 in benign and low-malignant lipomatous tumors. Int J Cancer.

[B25] Nilsson M, Mertens F, Höglund M, Mandahl N, Panagopoulos I (2006). Truncation and fusion of *HMGA2 *in lipomas with rearrangements of 5q32-->q33 and 12q14-->q15. Cytogenet Genome Res.

[B26] Dahlén A, Mertens F, Rydholm A, Brosjö O, Wejde J, Mandahl N, Panagopoulos I (2003). Fusion, disruption, and expression of *HMGA2 *in bone and soft tissue chondromas. Mod Pathol.

[B27] Livak KJ, Schmittgen TD (2001). Analysis of relative gene expression data using real-time quantitative PCR and the 2^-ΔΔCT^_T _Method. Methods.

[B28] Petit MM, Mols R, Schoenmakers EF, Mandahl N, Ven WJ Van de (1996). *LPP*, the preferred fusion partner gene of *HMGIC *in lipomas, is a novel member of the LIM protein gene family. Genomics.

[B29] Ashar HR, Tkachenko A, Shah P, Chada K (2003). *HMGA2 *is expressed in an allele-specific manner in human lipomas. Cancer Genet Cytogenet.

[B30] Crombez KR, Vanoirbeek EM, Ven WJ Van de, Petit MM (2005). Transactivation functions of the tumor-specific *HMGA2/LPP *fusion protein are augmented by wild-type *HMGA2*. Mol Cancer Res.

[B31] Von Ahsen I, Rogalla P, Bullerdiek J (2005). Expression patterns of the *LPP-HMGA2 *fusion transcript in pulmonary chondroid hamartomas with t(3;12)(q27~28;q14~15). Cancer Genet Cytogenet.

[B32] Tallini G, Dal Cin P, Rhoden KJ, Chiapetta G, Manfioletti G, Giancotti V, Fusco A, Berghe H Van den, Sciot R (1997). Expression of *HMGI-C *and *HMGI(Y) *in ordinary lipoma and atypical lipomatous tumors: immunohistochemical reactivity correlates with karyotypic alterations. Am J Pathol.

[B33] Tallini G, Vanni R, Manfioletti G, Kazmierczak B, Faa G, Pauwels P, Bullerdiek J, Giancotti V, Berghe H Van den, Dal Cin P (2000). *HMGI-C *and *HMGI(Y) *immunoreactivity correlates with cytogenetic abnormalities in lipomas, pulmonary chondroid hamartomas, endometrial polyps, and uterine leiomyomas and is compatible with rearrangement of the *HMGI-C *and *HMGI(Y) *genes. Lab Invest.

[B34] Kazmierczak B, Dal Cin P, Wanschura S, Borrmann L, Fusco A, Berghe H Van den, Bullerdiek J (1998). *HMGIY *is the target of 6p21.3 rearrangements in various benign mesenchymal tumors. Genes Chromosomes Cancer.

[B35] Dal Cin P, Kools P, Sciot R, De Wever I, Van Damme B, Ven W Van de, Berghe H Van den (1993). Cytogenetic and fluorescence in situ hybridization investigation of ring chromosomes characterizing a specific pathologic subgroup of adipose tissue tumors. Cancer Genet Cytogenet.

[B36] Pedeutour F, Forus A, Coindre JM, Berner JM, Nicolo G, Michiels JF, Terrier P, Ranchere-Vince D, Collin F, Myklebost O, Turc-Carel C (1999). Structure of the supernumerary ring and giant rod chromosomes in adipose tissue tumors. Genes Chromosomes Cancer.

[B37] Gisselsson D, Höglund M, Mertens F, Mitelman F, Mandahl N (1998). Chromosomal organization of amplified chromosome 12 sequences in mesenchymal tumors detected by fluorescence in situ hybridization. Genes Chromosomes Cancer.

[B38] Heidenblad M, Hallor KH, Staaf J, Jönsson G, Borg A, Höglund M, Mertens F, Mandahl N (2006). Genomic profiling of bone and soft tissue tumors with supernumerary ring chromosomes using tiling resolution bacterial artificial chromosome microarrays. Oncogene.

[B39] Fletcher CDM, Akerman M, Dal Cin P, de Wever I, Mandahl N, Mertens F, Mitelman F, Rosai J, Rydholm A, Sciot R, Tallini G, Berghe H Van den, Ven W Van de, Vanni R, Willén H (1996). Correlation between clinicopathological features and karyotype in lipomatous tumors. A report of 178 cases from the Chromosomes and Morphology (CHAMP) Collaborative Study Group. Am J Pathol.

[B40] Sandberg AA (2004). Updates on the cytogenetics and molecular genetics of bone and soft tissue tumors: lipoma. Cancer Genet Cytogenet.

[B41] Bassett MD, Schuetze SM, Disteche C, Norwood TH, Swisshelm K, Chen X, Bruckner J, Conrad EU, Rubin BP (2005). Deep-seated, well differentiated lipomatous tumors of the chest wall and extremities: the role of cytogenetics in classification and prognostication. Cancer.

[B42] Billing V, Mertens F, Domanski HA, Rydholm A (2008). Deep-seated ordinary and atypical lipomas: histopathology, cytogenetics, clinical features, and outcome in 215 tumours of the extremity and trunk wall. J Bone Joint Surg Br.

[B43] Italiano A, Cardot N, Dupré F, Monticelli I, Keslair F, Piche M, Mainguené C, Coindre JM, Pedeutour F (2007). Gains and complex rearrangements of the 12q13-15 chromosomal region in ordinary lipomas: the "missing link" between lipomas and liposarcomas?. Int J Cancer.

[B44] Italiano A, Bianchini L, Keslair F, Bonnafous S, Cardot-Leccia N, Coindre JM, Dumollard JM, Hofman P, Leroux A, Maniguené, Peyrottes I, Ranchere-Vince D, Terrier P, Tran A, Gual P, Pedeutour F (2008). *HMGA2 *is the partner of *MDM2 *in well-differentiated and dedifferentiated liposarcomas whereas *CDK4 *belongs to a distinct inconsistent amplicon. Int J Cancer.

[B45] Persson F, Olofsson A, Sjögren H, Chebbo N, Nilsson B, Stenman G, Åman P (2008). Characterization of the 12q amplicons by high-resolution, oligonucleotide array CGH and expression analyses of a novel liposarcoma cell line. Cancer Lett.

[B46] Sciot R, Akerman M, Dal Cin P, De Wever I, Fletcher CD, Mandahl N, Mertens F, Mitelman F, Rosai J, Rydholm A, Tallini G, Van den Berghe H, Vanni R, Willén H (1997). Cytogenetic analysis of subcutaneous angiolipoma: further evidence supporting its difference from ordinary pure lipomas: a report of the CHAMP Study Group. Am J Surg Pathol.

[B47] Cina SJ, Radentz SS, Smialek JE (1999). A case of familial angiolipomatosis with Lisch nodules. Arch Pathol Lab Med.

[B48] Ashar HR, Cherath L, Przybysz KM, Chada K (1996). Genomic characterization of human *HMGIC*, a member of the accessory transcription factor family found at translocation breakpoints in lipomas. Genomics.

[B49] Hebert C, Norris K, Scheper MA, Nikitakis N, Sauk JJ (2007). High mobility group A2 is a target for miRNA-98 in head and neck squamous cell carcinoma. Mol Cancer.

[B50] Wang T, Zhang X, Obijuru L, Laser J, Aris V, Lee P, Mittal K, Soteropoulos P, Wei JJ (2007). A micro-RNA signature associated with race, tumor size, and target gene activity in human uterine leiomyomas. Genes Chromosomes Cancer.

[B51] Sandberg R, Neilson JR, Sarma A, Sharp PA, Burge CB (2008). Proliferating cells express mRNAs with shortened 3' untranslated regions and fewer microRNA target sites. Science.

[B52] Rogalla P, Drechsler K, Frey G, Hennig Y, Helmke B, Bonk U, Bullerdiek J (1996). *HMGI-C *expression patterns in human tissues. Implications for the genesis of frequent mesenchymal tumors. Am J Pathol.

[B53] Weedon MN, Lettre G, Freathy RM, Lindgren CM, Voight BF, Perry JRB, Elliott KS, Hackett R, Guiducci C, Shields B, Zeggini E, Lango H, Lyssenko V, Timpson NJ, Burtt NP, Rayner NW, Saxena R, Ardlie K, Tobias JH, Ness AR, Ring SM, Palmer CN, Morris AD, Peltonen L, Salomaa V, Davey Smith G, Groop LC, Hattersley AT, McCarthy MI, Hirschhorn JN, Frayling TM (2007). A common variant of *HMGA2 *is associated with adult and childhood height in the general population. Nat Genet.

