# Supplementary material for: Expression levels of HMGA2 in adipocytic tumors correlate with morphologic and cytogenetic subgroups
Source: Mol Cancer. 2009 Jun 9;8:36. doi: 10.1186/1476-4598-8-36 (PMC2702300; doi:10.1186/1476-4598-8-36)
Supplement: Additional file 1 — Supplementary table. Clinical, cytogenetic, qRT-PCR, and FISH Data on 73 adipocytic tumors [file 1476-4598-8-36-S1.doc]

**Supplementary Table 1**. Clinical, cytogenetic, qRT-PCR, and FISH Data on 73 adipocytic tumors.

| **Diagnosis/**  **Case No.^a^** | **Sex/Age^b^** | **Location^c^** | **Karyotype^d^** | **qRT-PCR**  ***HMGA2*^e^**  **Log10 value** | | **FISH**  ***HMGA2*^f^** |
| --- | --- | --- | --- | --- | --- | --- |
|  |  |  |  | **Exons 1-2** | **Exons 4-5** |  |
| **Lipoma with**  **t(3;12)** |  |  |  |  |  |  |
| 1 | M/49 | Back | 46,XY,t(3;12)(q27;q13) | 3.15 | 0.83 |  |
| 2 | F/47 | Trunk | 46,XX,t(3;12)(q27;q14) | 3.08 | 0.74 |  |
| 3 | M/39 | Thigh | 46,XY,t(3;12)(q27;q15) | 3.00 | 0.35 |  |
| 4^g^ | F/30 | Shoulder | 46,XX,t(3;12)(q27;q15) | 2.97 | 1.49 |  |
| 5 | F/47 | Thorax | 46,XX,t(3;12)(q27;q14) | 2.72 | 1.04 |  |
| **Lipoma with**  **t(5;12)** |  |  |  |  |  |  |
| 6 | F/88 | Arm | 46,XX,t(5;12)(q33;q15) | 3.61 | 3.47 | Normal |
| 7 | F/68 | Thigh | 46,XX,t(5;12)(q32;q14)/46,idem,  add(2)(p14),add(4)(q21),der(18)  t(4;18)(q21;q?21) | 3.15 | 3.15 | Normal |
| 8 | M/70 | Leg | 46,XY,t(5;12)(q33;q14)/46,idem,  t(1;6)(p36;p23) | 3.13 | 3.13 |  |
| 9^h^ | M/60 | Neck | 46,XY,ins(12;5)(q15;q33q13) | 2.96 | -0.84 | Split |
| 10^g^ | F/44 | Thigh | 46,XX,t(5;12)(q33;q14) | 2.74 | 2.73 | Relocated |
| **Lipoma with**  **der(12q13-15)** |  |  |  |  |  |  |
| 11^g^ | F/61 | Shoulder | 46,XX,inv(12)(q14q24) | 2.79 | -1.29 | 3’deletion |
| 12 | M/67 | Neck | 46,XY,t(2;12)(q37;q14) | 2.77 | 0.11 | Split |
| 13^g^ | M/75 | Thigh | 46,XY,t(1;12)(p32;q15) | 2.65 | 2.71 | Split |
| 14 | F/66 | Arm | 46,XX,t(10;12)(q22;q15) | 2.42 | 0.46 | Split |
| 15 | F/26 | Thigh | 46,XX,t(1;12;17)(p35;q15;q21) | 2.12 | 2.20 |  |
| **Lipoma with**  **variant aberration** |  |  |  |  |  |  |
| 16 | F/42 | Thigh | 46,XX,t(3;17)(p25;q11) | 2.17 | -0.64 |  |
| 17^g^ | M/54 | Leg | 46,XY,t(10;22)(q22;q13)/  45,idem,-20 | 2.04 | 2.08 |  |
| 18^g^ | F/42 | Arm | 46,XX,der(4)t(4;15)(p16;q22),t(5;9)(q22;q32),ins(8;13)(q24;q34q14),  add(15)(q15),add(16)(q13),der(20)  t(16;20)(q13;q12) | 1.85 | 1.96 | Normal |
| 19 | F/19 | Neck | 47,XX,+i(8)(q10) | 1.80 | -0.42 | 3’deletion |
| 20 | F/36 | Groin | 46,XX,inv(3)(p21q29) | -0.88 | -1.05 | Normal |
| **Lipoma with der(6p21-22)** |  |  |  |  |  |  |
| 21^g^ | F/33 | Shoulder | 46,XX,t(3;6)(q24;p22) | 2.94 | 0.92 | Normal |
| 22 | M/52 | Arm | 46,XY,t(3;6;12)(q27;p21;q22)/  46,idem,inv(9)(p13q34) | 0.29 | -0.39 |  |
| 23 | M/53 | Thigh | 46,XY,t(1;6)(p32;p21) | 0.13 | -0.18 |  |
| 24 | F/61 | Hip | 46,XX,inv(6)(p21q21) | -0.38 | -0.50 |  |
| 25 | F/68 | Thigh | 46,XX,t(1;6)(p32;p21) | -0.40 | -0.65 |  |
| **Lipoma with del(13q)** |  |  |  |  |  |  |
| 26^g^ | M/47 | Thigh | 46,XY,del(13)(q12q22) | 2.89 | -0.05 |  |
| 27^g^ | M/63 | Back | 46,XY,del(13)(q14q22) | 1.27 | 1.00 |  |
| 28 | M/60 | Thigh | 46,XY,t(12;20)(q13;q11),  del(13)(q12q22-31) | 0.74 | 0.78 |  |
| 29 | F/44 | Shoulder | 45,XX,del(6)(q21),del(13)(q14),-16 | -0.19 | -0.27 |  |
| 30 | M/49 | Arm | 46,XY,der(6)t(6;13)(p2?3;q3?2),  der(13)t(6;13)(p2?3;q14) | -0.92 | -1.03 |  |
| **Lipoma with ring(s)** |  |  |  |  |  |  |
| 31 | M/54 | Thigh | 46-48,XY,+1-2r,+mar | 3.23 | -0.63 |  |
| 32^i^ | F/43 | Thigh | 47-48,XX,+1-2mar | 2.96 | 2.84 |  |
| 33 | F/79 | Shoulder | 47,XX,+r/47-48,XX,+1-2r/  47-49,XX,+1-3r | 2.80 | 2.98 |  |
| 34 | M/32 | Shoulder | 45-47,XY,-4,-14,+1-2r,+mar | 2.69 | 2.64 |  |
| 35 | F/60 | Thigh | 47,XX,+r/47,XX,+mar | 2.45 | -0.53 |  |
| **Angiolipoma** |  |  |  |  |  |  |
| 36^g^ | F/59 | Arm | ND | 1.64 | 1.67 |  |
| 37^g^ | F/59 | Thigh | ND | 1.42 | 1.54 | Normal |
| 38^g^ | M/43 | Arm | 46,XY | 1.10 | 1.03 |  |
| 39^g^ | F/36 | Trunk | 46,XX | 0.68 | 0.61 | Normal |
| 40^g^ | F/44 | ? | 46,XX | 0.67 | 0.73 |  |
| 41^g^ | F/28 | ? | 46,XX | ND | ND |  |
| 42^g^ | M/56 | Leg | 46,XY | ND | ND |  |
| 43^g^ | M/51 | Thigh | 46,Y,t(X;2)(p22;p12) | ND | ND |  |
| **Spindle cell lipoma** |  |  |  |  |  |  |
| 44 | M/67 | Shoulder | 46,XY,del(13)(q14) | 1.76 | -0.51 |  |
| 45 | M/78 | Neck | 58,XXY,+Y,-1,-2,-4,-6,-7,+8,-9,  -10,-12,-13,-14,-16,-17,-22 | 0.33 | 0.15 |  |
| 46 | F/35 | Neck | 44,XX,add(4)(p11),-9,add(10)  (q22),-13,-16,add(17)(q11),der(17)  t(9;17)(p13;p13),add(22)(q11),+mar/88,idemx2 | -0.09 | -0.21 |  |
| 47 | M/63 | Back | 43-45,XY,?der(2)t(2;13)(q13;q14),  del(6)(q13),?-13,-16,+mar | -0.46 | -0.46 |  |
| 48 | M/76 | Arm | 46,XY,inv(9)(p11q12)c,  del(13)(q12q21) | -0.55 | -0.85 |  |
| **Hibernoma** |  |  |  |  |  |  |
| 49^g^ | F/34 | Thigh | ND | 1.60 | 1.36 |  |
| 50 | F/25 | Thigh | 46,XX,t(6;14;11)(p21;q11;q13) | -0.39 | -0.50 |  |
| 51 | M/16 | Thigh | ND | -0.53 | -0.71 |  |
| 52 | F/51 | Thigh | 46,XX,t(11;17;12)(q13;q12;p13) | -0.54 | -0.66 |  |
| 53 | M/39 | Arm | 46,XY,t(2;11)(p21;q13),ins(17;12)  (q21;p11p13)/46,XY,der(11)t(2;11)  (p21;q13),del(12)(p11),add(17)(q21) | -0.91 | -1.21 |  |
| **Atypical lipoma** |  |  |  |  |  |  |
| 54 | M/91 | Thigh | 46,X,-Y,+r | 4.59 | 0.05 | Split &  Amplified |
| 55 | M/86 | Thigh | 47-48,XY,add(22)(p13),+1-2r/  47,XY,add(22),+mar | 4.31 | 0.58 | Split &  Amplified |
| 56 | M/55 | Thigh | 47-50,XY,+1-4r | 3.90 | -0.77 |  |
| 57 | M/96 | ? | 46-47,X,-Y,+1-2r | 3.61 | -0.04 | Split &  Amplified |
| 58 | M/55 | Thigh | 47,XY,+r | 3.47 | 3.30 |  |
| 59 | M/79 | Thigh | 51-53,XY,+5-7r/  99-105,XXYY,+7-13r | 3.46 | 0.79 |  |
| 60 | F/78 | Thigh | 47-48,XX,+r,+mar/47-48,idem,  +9,-18 | 3.30 | 3.32 |  |
| 61 | F/87 | Thigh | 47,XX,+r | 3.14 | -0.75 | Split &  Amplified |
| 62 | M/68 | Thigh | 47-51,XY,+1-3r,+1-2mar | 3.11 | 3.23 |  |
| 63 | F/68 | Thigh | 47-48,XX,+1-2r/49,XX,+2r,+mar/  49-50,XX,der(3)t(3;13)(p25;q12),  -13,+3-4r,+mar/90-92,XXXX,+2-3r | 2.13 | 2.22 |  |
| **WDLS** |  |  |  |  |  |  |
| 64 | M/54 | Retroper | 47,XY,+mar | 4.11 | 1.95 |  |
| 65 | M/60 | Retroper | 45-48,XY,+1-3r/84-91,XXYY,+1-6r | 4.04 | 4.13 |  |
| 66 | M/63 | Retroper | 47,XY,+r | 3.55 | 0.35 |  |
| 67 | F/79 | Retroper | 44-46,XX,+2r/90-94,idemx2 | 2.89 | 2.39 |  |
| 68 | F/72 | Retroper | 43-47,XX,+r | 2.33 | 2.32 |  |
| **MLS** |  |  |  |  |  |  |
| 69 | F/54 | Thigh | 46,XX,t(12;16)(q13;p11) | 0.44 | -0.42 |  |
| 70 | F/43 | Thigh | 47,XX,+8,t(12;16)(q13;p11) | 0.41 | 0.13 |  |
| 71 | M/52 | ? | 46,XY,+1,der(1;16)(q10;p10),  t(7;14)(p14;p13),t(12;16)(q13;p11) | 0.11 | -0.27 |  |
| 72 | M/60 | Leg | 46,XY,t(12;13;16)(q13;q12;p11) | 0.05 | 0.19 |  |
| 73 | M/61 | Thigh | 46,XY,t(12;16)(q13;p11) | 0.03 | -0.56 |  |

^a^WDLS = well-differentiated liposarcoma; MLS = myxoid liposarcoma.

^b^F = female; M = male; Age in years at diagnosis.

^c^Retroper = retroperitoneal; ? = unknown location.

^d^The karyotypes of cases 1-9, 16-20, 22-24, 26-35, 43 and 46 have been published previously (Bartuma et al., 2007; Dahlén et al., 2003a; Heim et al., 1987; Mandahl et al., 1994; Nilsson et al., 2006). ND = not determined.

^e^qRT-PCR = quantitative reverse transcriptase polymerase chain reaction. Expression levels are given as Log10 values. Conventional RT-PCR data on the expression of full-length and truncated *HMGA2* in cases 16-20 have been reported before (Bartuma et al., 2007). ND = not determined.

^f^FISH = fluorescence in situ hybridization. The FISH results on cases 6, 7, and 18-20 have been reported before (Bartuma et al., 2007; Nilsson et al., 2006).

^g^Analyzed with genomic PCR and RT-PCR regarding the status of the 3’ UTR of *HMGA2*.

^h^One lipoma with ins(12;5) was included among the t(5;12) cases.

^i^The markers found were so-called giant markers, i.e., rod-shaped equivalents of rings.
