# Supplementary figures and images for: Expression levels of HMGA2 in adipocytic tumors correlate with morphologic and cytogenetic subgroups
Source: Mol Cancer. 2009 Jun 9;8:36. doi: 10.1186/1476-4598-8-36 (PMC2702300; doi:10.1186/1476-4598-8-36)

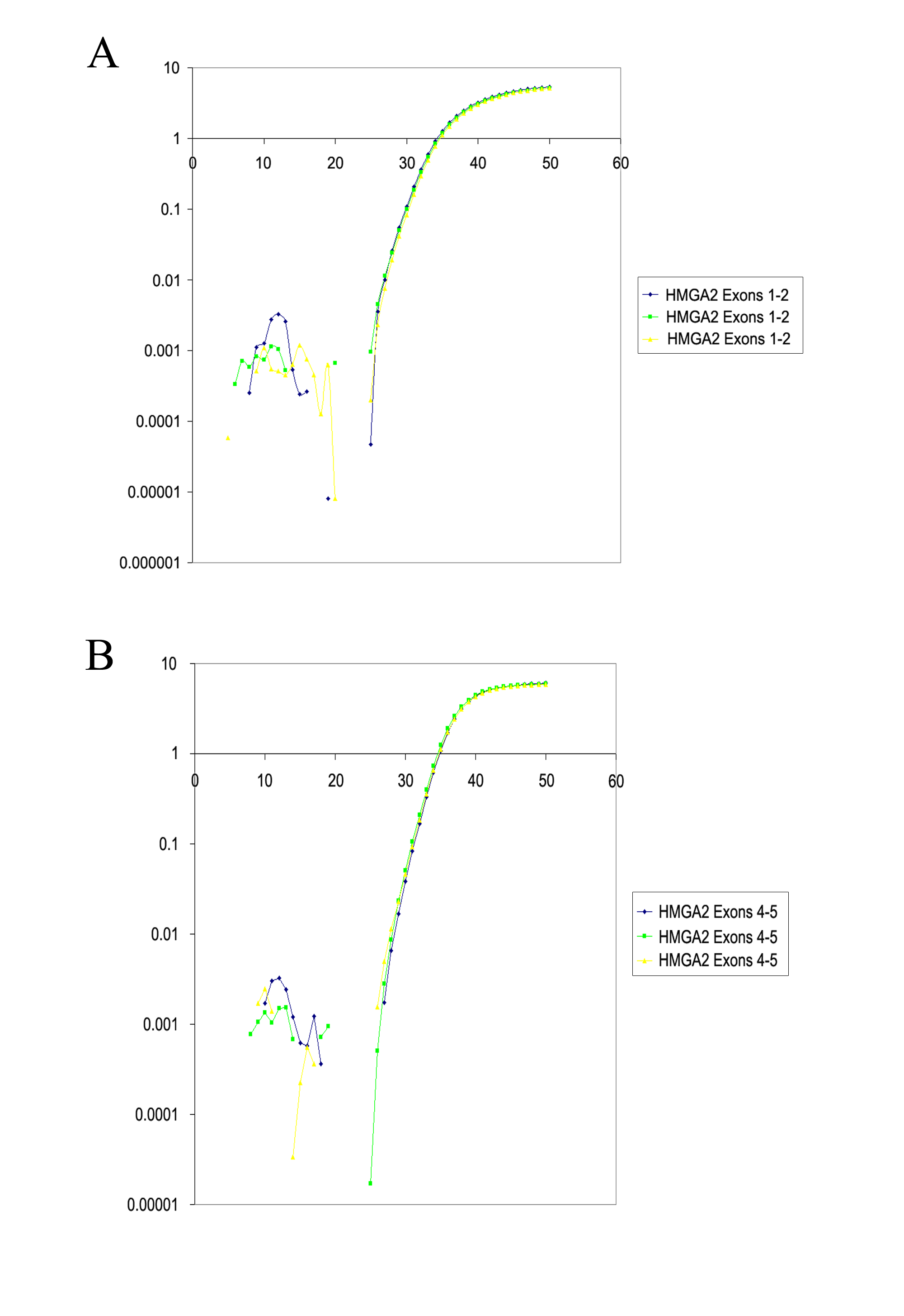

Supplement: Additional file 2 — Expression pattern of HMGA2 exons 1–2 and 4–5 in human adipose tissue. A) exons 1–2 and B) exons 4–5 of HMGA2 in human adipose tissue [Ambion's total Human RNA] showing a late expression pattern, starting after 35 cycles in both amplifications. Three replicates were run. [file 1476-4598-8-36-S2.tiff]

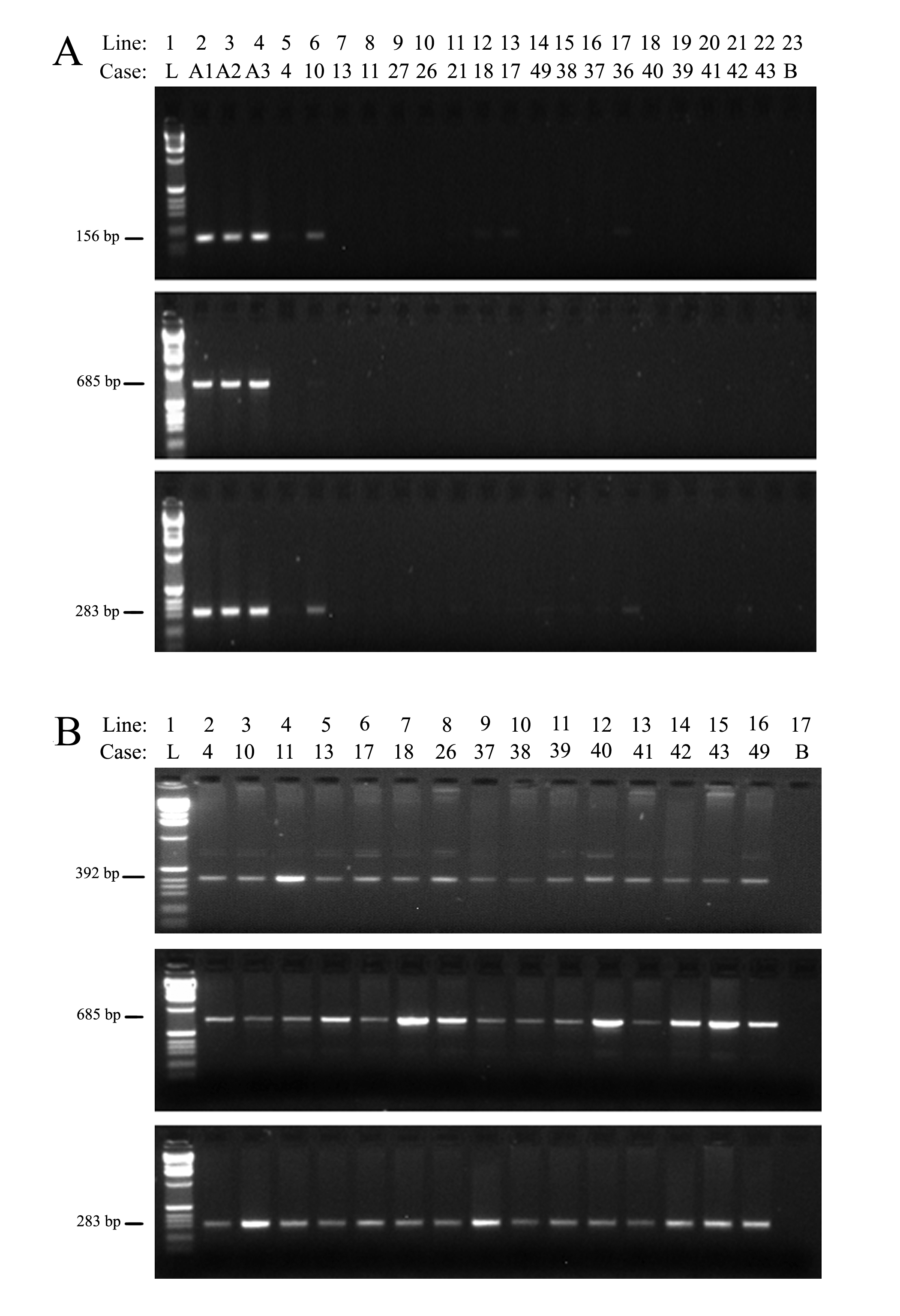

Supplement: Additional file 4 — RT-PCR and genomic PCR for the 3'UTR of HMGA2. RT-PCR and genomic PCR for the 3'UTR of HMGA2 in 3 amniocytic cell cultures and 18 adipocytic tumors. [file 1476-4598-8-36-S4.tiff]

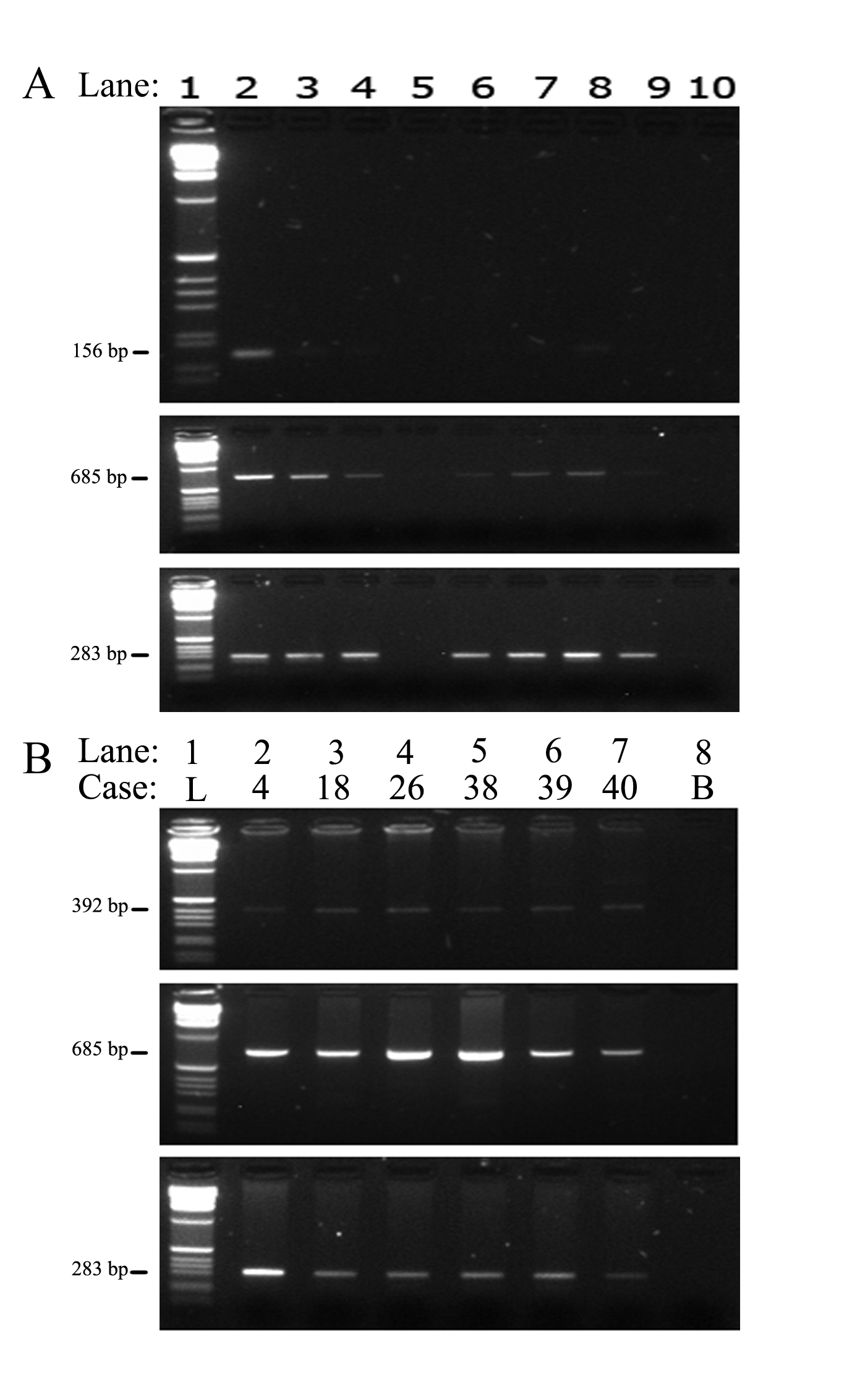

Supplement: Additional file 5 — RT-PCR and genomic PCR for the 3'UTR of HMGA2. RT-PCR analysis of the 3'UTR of HMGA2 in fetal tissues and genomic PCR analysis of the 3'UTR of HMGA2 in DNA from peripheral blood from six of the patients with lipomatous tumors. [file 1476-4598-8-36-S5.tiff]
