# Supplementary material for: Expression levels of HMGA2 in adipocytic tumors correlate with morphologic and cytogenetic subgroups
Source: Mol Cancer. 2009 Jun 9;8:36. doi: 10.1186/1476-4598-8-36 (PMC2702300; doi:10.1186/1476-4598-8-36)
Supplement: Additional file 3 — Supplementary table. Primers for PCR [file 1476-4598-8-36-S3.doc]

**Supplementary Table 2.** Primers for PCR.

Name^1^ Sequence Nucleotides PCR Products (bp)

**Region 1**

HMGA2-1066F1_A_ 5’-ACAAGTTGTTCAGAAGAAGCCTG 1066-1088

HMGA2-1222R1_A_ 5’-AGTGGAAAGACCATGGCAATAC 1243-1222 156

HMGA2-11802F1_B_ 5’-CATAATGTGCTGTGGAAACAGG 11802-11823

HMGA2-12194R1_B_ 5’-ATTGTTAAGCTGTGTCCCCTGT 12215-12194 392

**Region 2**

HMGA2-2204F2 5’-CTTTTCATTGTGTATCAGTTTCCA 2204-2227

HMGA2-2889R2 5’-ATAGTTTTGACTAGGGTTAGCTGC 2912-2889 685

**Region 3**

HMGA2-3527F3 5’-TTTAGAAACCTCATTGGCCAGC 3527-3548

HMGA2-3810R3 5’-TCCAGTCTTATGTAGCTGCGACC 3832-3810 283

^1^Primer orientation: F = forward, R = reverse. All sequences are retrieved from NM_003483.4, except primers HMGA2-11802F1_A_ and HMGA2-12194R1_A_ which come from chromosome 12: 64631440+64643321, http://genome.ucsc.edu/.
